# Immortalization of Human Neural Stem Cells with the c-Myc Mutant T58A

**DOI:** 10.1371/journal.pone.0003310

**Published:** 2008-10-02

**Authors:** Lidia De Filippis, Daniela Ferrari, Laura Rota Nodari, Bruno Amati, Evan Snyder, Angelo Luigi Vescovi

**Affiliations:** 1 Università degli Studi Bicocca-Millan, Milan, Italy; 2 Istituto Oncologico Europeo, Milano, Italy; 3 Stem Cell and Regeneration Program, Burnham Institute for Medical Research, La Jolla, California, United States of America; Katholieke Universiteit Leuven, Belgium

## Abstract

Human neural stem cells (hNSC) represent an essential source of renewable brain cells for both experimental studies and cell replacement therapies. Their relatively slow rate of proliferation and physiological senescence in culture make their use cumbersome under some experimental and pre-clinical settings. The immortalization of hNSC with the v-myc gene (v-IhNSC) has been shown to generate stem cells endowed with enhanced proliferative capacity, which greatly facilitates the study of hNSCs, both in vitro and in vivo. Despite the excellent safety properties displayed by v-IhNSCs – which do not transform in vitro and are not tumorigenic in vivo – the v-myc gene contains several mutations and recombination elements, whose role(s) and effects remains to be elucidated, yielding unresolved safety concerns. To address this issue, we used a c-myc T58A retroviral vector to establish an immortal cell line (T-IhNSC) from the same hNSCs used to generate the original v-IhNSCs and compared their characteristics with the latter, with hNSC and with hNSC immortalized using c-myc wt (c-IhNSC). T-IhNSCs displayed an enhanced self-renewal ability, with their proliferative capacity and clonogenic potential being remarkably comparable to those of v-IhNSC and higher than wild type hNSCs and c-IhNSCs. Upon growth factors removal, T-IhNSC promptly gave rise to well-differentiated neurons, astrocytes and most importantly, to a heretofore undocumented high percentage of human oligodendrocytes (up to 23%). Persistent growth-factor dependence, steady functional properties, lack of ability to generate colonies in soft-agar colony-forming assay and to establish tumors upon orthotopic transplantation, point to the fact that immortalization by c-myc T58A does not bring about tumorigenicity in hNSCs. Hence, this work describes a novel and continuous cell line of immortalized human multipotent neural stem cells, in which the immortalizing agent is represented by a single gene which, in turn, carries a single and well characterized mutation. From a different perspective, these data report on a safe approach to increase human neural stem cells propagation in culture, without altering their basic properties. These T-IhNSC line provides a versatile model for the elucidation of the mechanisms involved in human neural stem cells expansion and for development of high throughput assays for both basic and translational research on human neural cell development. The improved proclivity of T-IhNSC to generate human oligodendrocytes propose T-IhNSC as a feasible candidate for the design of experimental and, perhaps, therapeutic approaches in demyelinating diseases.

## Introduction

Neural stem cells (NSC) are pivotal players in the development of the central nervous system development, maintenance and repair [1]–[7]. As such, they hold great potential in the areas of drug discovery, cell therapy, replacement and cell-mediated gene therapy.

Human neural stem cells (hNSCs) have been shown to provide a plentiful, renewable source of neural for cell replacement. Notwithstanding, as expected for somatic stem cells, following a finite number of cell divisions in culture, hNSCs will eventually undergo growth arrest and senescence. This limits their exploitation in the field of biotechnology and in pharmacological studies relying on large scale, high-throughput assays. In this perspective, an alternative source of human brain cells, bearing all the features of wild type hNSCs and possessing unlimited expandability, would be of immense value for modelling studies in neuroscience, drug discovery and cell therapy.

We and others have shown how oncogene-mediated immortalization of human neural precursors/stem cells provides an initial attempt to overcome these limitations, leading to the establishment of immortal hNSC lines [5], [8]. Despite these results, it is highly desirable to obtain immortalized neural stem cell lines, in which the immortalizing agent is better characterized and predictable in its effects, so that the immortalized lines will mimic the behavior of normal hNSCs as closely as possible also, tentatively, to be used in a cell therapy context.

We have recently described the immortalization of wild type hNSCs with v-myc, resulting in the establishment of a stable neural stem cell line (v-IhNSC) endowed with the ability to originate mature functional neurons and conspicuous amounts of oligodendrocytes in vitro [9]. This line never showed any sign of transformation, retained unchanged functional features, overlapping those of its wild-type counterpart, and strict growth factor dependence. Given the well documented oncogenic potential of v-myc [10], [11] and the fact that its regulatory mechanisms remain to be fully elucidated, we decided to improve this strategy by looking for an immortalizing, well-characterized gene which was as similar as possible to its wild-type gene while, at the same time, possessing most of the advantages of v-myc.

In this perspective, we argued that a candidate immortalizing gene, containing a single, specific mutation, would represent an optimal candidate to obtain novel IhNSCs. To accomplish this goal, we decided to attempt immortalization of wild type hNSCs by means of a c-mycT58A expressing retroviral vector [12] and compared the properties of the resultant immortalized cells (T-IhNSC) to that of their normal counterparts, as well as to those of both v-myc (v-IhNSC) and c-myc wt (c-IhNSC) immortalized human neural stem cells [9]. The T58A c-myc mutant was chosen as a candidate immortalizing agent in considering that the intracellular levels of c-myc protein play a critical role in proliferation and that the stability and accumulation of c-myc are regulated by multiple Ras effector pathways. Since the phosphorylation of the Thr58 residue in c-myc is important for its degradation – for the former acts as a recognition site for the ubiquitin ligase Fbw7 – the mutation of Thr58 to alanine found in T58A c-myc results in a more stable c-myc protein, with a consequent increase in its intracellular levels, reduced apoptosis and increased proliferation activity [11], [13]–[22]. Accordingly, several v-myc genes from transforming retroviruses harbor mutations at the Thr58 residue [23], [24]. Similarly, the c-myc gene translocated in Burkitt's lymphoma, frequently carries a mutation at the Thr58 residue [15], [17], [19]–[21] and shows reduced pro-apoptotic function in both cell culture assays and animals [11], [13], [16], [19], [20].

In this study, we report for the first time that T58A c-myc successfully immortalizes hNSCs and that these T-IhNSCs proliferate faster than their wild-type counterpart and c-IhNSC immortalized cells, but less than v-IhNSCs. Notably, T-IhNSC cells rapidly stop dividing upon removal of growth factors and reach terminal differentiation within 14 days in vitro, similar to normal non-immortalized cells (hNSC). Moreover, their differentiation profile appears to encompass a unique ability to generate an extremely high percentage of oligodendrocytes and of neuronal cells, in a fashion comparable to that v-IhNSCs [9]. Thus, by this work we introduce a novel cell line of human neural stem cells, which have been immortalized by a gene that carries a single, well characterized mutation and putative mechanism of action. In addition, these T-IhNSCs bear similar, useful functional features – multipotentiality, stability, neuronal and oligodendroglia differentiation and improved growth rate – as compared to previously established v-IhNSCs, but bear a significantly lesser probability of tumorigenic transformation.

## Materials and Methods

### Propagation and differentiation of neural stem cells

The parental human neural stem cells (hNSCs) used in this study have been reported previously [25]. Briefly, cells were derived from the diencephalic and telencephalic brain regions of a 10.5-week gestational-age aborted Caucasian human fetus and cultured in the presence of 20 ng/ml of human recombinant EGF and and 10 ng/ml of FGF2 (Tebubio, Milan, Italy), in NS-A basal serum-free medium (Euroclone, Irvine, Scotland) containing 2 mM L-glutamine, 0.6% glucose, 9.6 ug/ml putrescine, 6.3 ng/ml progesterone, 5.2 ng/ml sodium selenite, 0.025 mg/ml insulin, 0.1 mg/ml transferrin (sodium salt, grade II; Sigma) [25].

To induce hNSC differentiation, individual spheres were mechanically dissociated and cells were transferred at a density of 2.5×10ˆ4 cells/cm^2^ onto matrigel (Sigma)-coated chamber-slides in the presence of 20 ng/ml FGF2. After 72 hrs, cultures were shifted to NS-A control medium (CM) containing 2% fetal calf serum (FCS) and grown for two weeks. This treatment resulted in a mixed culture of neural cells containing astrocytes, neurons and oligodendrocytes [25]. Afterwards, they were fixed and processed for immunostaining at appropriate time-points.

### Retroviral infections

Retroviral constructs utilized are based on p-Babe, a replication-defective retroviral vector conferring resistance to neomycin. Constructs encoding either c-myc or c-myc T58A were used to transduce hNSCs at 17 passages from the primary culture. A mock vector was used as a control. v-IhNSCs were generated by immortalization with the v-myc gene [9].

Parental hNSCs were first expanded with growth factors (EGF+FGF2) as neurospheres up to passage 17. After mechanical dissociation, the cells were cultured in EGF+FGF2 containing medium for 24 hours in order to obtain a culture enriched in stem cells. Retroviral transduction was then performed by a repeated-infection procedure: a pretransduction period in the presence of EGF and FGF2 (days 0–2) was used to obtain an adherent culture growing on a laminin-coated plastic substrate while division was stimulated by growth factors. Retroviral vector supernatant (GF medium) supplemented with polybrene (8 ug/ml) was filtered and then added to the cells. Three rounds of infection were performed in a period of 36 hrs, after which the cells were returned to fresh GF-containing medium and expanded as neurospheres (in suspension) for 9 passages. Selection of neomycin-resistant transduced cells was carried out with 1200 ug/ml G418. Aliquots of these non-clonal cell lines were cryopreserved in GF culture medium containing 10%DMSO.

To induce IhNSC differentiation, individual spheres were mechanically dissociated and were transferred at a density of 1×10ˆ4 cells/cm^2^ onto laminin (Roche)-coated glass coverslips in the presence of 20 ng/ml FGF2. After 72 hrs, cultures were shifted to serum-deprived NS-A control medium (CM) and grown for up to two weeks to obtain a mixed culture of neural cells containing astrocytes, neurons and oligodendrocytes [26].

### Clonal analysis

IhNSC neurospheres were mechanically dissociated (>200 extrusion cycles through a p200, Gilson yellow tip pipette) to yield a single cell suspension. Cells were plated by serial, limiting dilution [27] to obtain a statistical distribution of <1 cell/well in 96 well plates (BD Biosciences), in complete growth medium containing EGF and FGF-2. Single wells were scored by direct visualization under a phase contrast microscope and only those containing one cell per well were taken into account. Slightly less than 40% of the wells containing single cells gave rise to a clonal cell line. Three of these clones were chosen as representative and characterized by growth curve analysis and differentiation assay, yielding comparable results.

### Clonogenic assay

Neurospheres from different cultures were dissociated and single cells were plated at decreasing densities in 24 well plates (BD Biosciences) precoated with poly-lisine in complete medium containing EGF and FGF-2. IhNSC cells were plated at 5000, 2500 and 1000 cells per well. After 4 days in vitro, the fraction of wells positive for neurosphere formation was quantified. The data are presented as the percentage of neurospheres of the total number of plated cells: every value corresponds to the the average of three separate coverslips for each density. The density of 2500 cells per well was chosen as representative, as similar results came from the other two cell densities. The experiment was repeated twice [27].

### Viability assay

Upon mechanical dissociation of expanding neurospheres from c-IhNSC, T-IhNSC and v-IhNSC cultures, 200.000 cells from each culture were plated in separate T25 flasks containing 5 ml of basal culture medium. Three T25 flasks were simultaneously plated for each culture and live cells were counted at 24, 48 and 72 hrs by trypan blue staining in a Burker chamber. The experiment was repeated twice.

### Immunocytochemistry

Cultures were fixed in freshly prepared, buffered 4% paraformaldehyde. After blocking with 10% normal goat serum, the cultures were incubated overnight at 4°C with the following antibodies (mAb, monoclonal; pAb, polyclonal): β-tubulin isotypeIII (mAb, MMS-435P Babco, 1∶400), glial fibrillar acidic protein (GFAP pAb, Dako 1∶400), galactocerebroside C (Gal-C, mAb MAB345 Chemicon 1∶100), O4 (mAb, MAB342 Chemicon 1∶100), tyrosine-hydroxylase (TH mAb, Novocastra NCL-TH, 1∶100), gamma aminobutirric acid (GABA pAb, Sigma, 1∶1000), glutamatergic acid (GLUTAMATE pAb, Sigma 1∶3000), microtubule-associated protein 2 (MAP2 mAb, Chemicon 1∶400), KI67 nuclear antigen (pAb, Novocastra NCL-Ki67p 1∶1000), vimentin (mAb, Chemicon 1∶400), MAP5 (mAb Immunological Science 1∶400).

After removal of the primary antibodies and repeated washes with PBS, cultures were incubated for 45 minutes at room temperature with secondary antibodies Alexa 488 (A11008 Molecular Probe, anti-rabbit 1∶1000) and/or Alexa 546 (A11030 Molecular Probe, anti-mouse 1∶1000). Samples were then coloured with dapi (0,3 µg/ml, Roche) for nuclear staining and rinsed with PBS for mounting and analyses. Microscopic examination and photography of specimens were performed with a Zeiss Axiovert 200 direct epifluorescence microscope.

Data are reported as percentages of labeled cells over the total number of nuclei +/− the standard error of the mean (SE). An average total amount of 3000 cells (identified by Dapi nuclear staining) was counted randomly from 2 coverslips per condition in each experiment. Each value represents the average of three independent experiments.

## Results

### Transduction of hNSCs with c-mycT58A enhances proliferation and expansion

**Figure 1:**
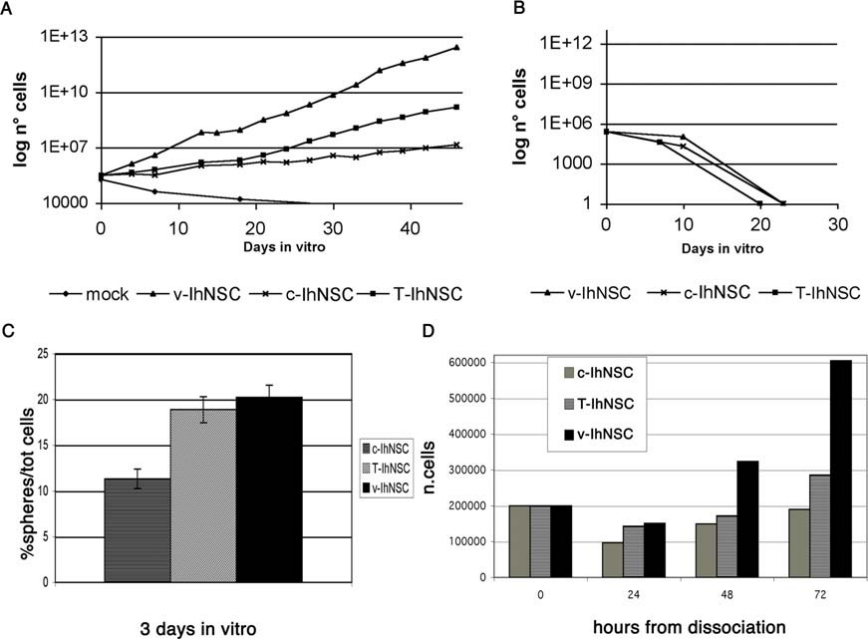
Analysis of long-term proliferation in T58A stem cells. A) Growth curve of c-IhNSC, T-IhNSC, v-IhNSCs and mock cultures in the presence of EGF and FGF2 in the culture medium. The slopes of the curves show how much higher the proliferation rate of IhNSCs cells is than hNSC cells. At each passage, 200.000 cells were plated and counted when they originated neurospheres. B) Growth curve of c-IhNSC, T-IhNSC, v-IhNSCs in absence of growth factors. Cells could not be expanded using these conditions. C) Clonogenic assay of c-IhNSC, T-IhNSC, v-IhNSCs. Values correspond to the average value of two experiments (three samples per experiment). D) Viability assay of c-IhNSC, T-IhNSC cells, IhNSCs at 24, 48, 72 hours from dissociation of neurospheres and plating of 200.000 cells.

Previous data from Cartwright et al. [12] showed that sustained expression of c-myc T58A in murine ES cells elicits increased self-renewal and maintenance of pluripotency. To test if c-myc T58A could be used as an immortalizing gene, hNSCs established previously from a human fetus forebrain at 10.5 pcw (parental cells; [25]) were transduced with a retroviral construct expressing wild type c-myc or c-myc T58A [17]. Transgene expression was confirmed by RT-PCR (not shown). After selection in neomycin-containing medium, T58A (T-IhNSC) and c-myc (c-IhNSC) lines were found to proliferate and to form neurospheres in the presence of EGF and FGF2, similar to their parental cells [25]. However, while c-IhNSC expressed an unchanged growth rate kinetic, the growth of T-IhNSC increased significantly, approaching an estimated doubling time of 4–5 days (Fig. 1 A) as compared to the standard 8–10 days of their parental cells [25]. The increased growth rate of T-IhNSCs was only slightly lower than the 3–4 days observed in the same cells previously immortalized using v-myc (v-IhNSCs)[9]. In turn, mock-infected cells underwent senescence after few (2–3) passages from antibiotic selection, re-emphasizing the well-known sensitivity of wild type hNSCs cells to extensive and somewhat harsh transduction manipulations.

As hoped, immortalized T-IhNSC cells did retain strict growth factor dependence, shown by the fact that, upon removal of EGF and bFGF from the growth medium, they underwent loss of proliferation ability (Fig. 1B). In addition to their proliferative capacity, an essential property of *bona fide* stem cells is their self-renewal potential, which can be demonstrated in neural stem cell cultures by observing a linear cell growth kinetic under the appropriate neurosphere assay conditions and in long-term cultures [28]. Since Immortalization is expected to perpetuate the ability of stem cells to self-renew [29], we subjected T-IhNSCs neurospheres to long-term, sequential passaging as bulk cultures, in order to determine their extensive self-renewal capacity and related steady growth properties. As expected T-IhNSCs were easily maintained and steadily expanded in culture for up to at least 120 passages, differently from their parental hNSCs, which undergo spontaneous senescence after 45–50 passages [25]. T-IhNSCs' growth rate was stable over passaging and so was their strict growth factor dependence.

To obtain a direct evidence of the improved self-renewal capacity of T-IhNSC we performed a clonogenic, neurosphere assay. As expected, the clonogenic index of T-IhNSC cultures after dissociation (18.88±1.43 S.E.% of the total of plated cells) was somewhat similar to that of v-IhNSC (20.22±1.33 S.E.%), but significantly increased with respect to c-IhNSC (11.33±1.07 S.E.%) or to parental cells [30], [31] (Fig. 1C).

These results were integrated by a viability assay, which demonstrated comparable percentages of surviving cells in T-IhNSC and v-IhNSC clonogenic cultures, at least 50% higher as compared to c-IhNSC cells (Fig. 1D) or parental cells [30].

Altogether, these findings demonstrate that by T58 c-myc transduction of hNSCs, we have generated a novel immortalized hNSCs cell line endowed with rapid proliferation capacity, strict and increased and extended self-renewal ability.

### Cell cycle analysis

To investigate whether enhanced cell proliferation in T-IhNSC cells was due to an increased proliferating cell fraction or to shortening of the duration of the cell cycle, we analysed cell distribution throughout cell cycle phases. Cytofluorimetric analysis showed that the average T-IhNSC and c-IhNSC cell cycle times were comparable (approximately 50–55 hrs), whereas v-IhNSC entertained a significantly faster cell cycle (about 40 hrs). Of note, over 25% of cells were actively engaged in the S phase in all of the three immortalized cultures, as compare to about 2.5% in parental cell cultures (Figure S1). These results point to the conclusion that T-IhNSC expand faster in number than c-IhNSC cells due to a higher percentage of viable proliferating cells being produced at each generation (Fig. 1C, D). Conversely, their lower rate of amplification in culture as compared to v-IhNSC, is likely due to a longer cell cycle.

Taken together, these findings show that c-mycT58A-mediated immortalization of cultured hNSCs leads to the establishment of immortalized neural cells endowed with an extensive and rapid proliferation capacity, which retain functional key characteristic of their parental cells, such as the typical steady growth profile and growth factor-dependence observed in parental cells.

### T58A cells are multipotent

To confirm that the immortal T-IhNSCs lines that we had established did, in fact, bear the features of hNSCs, we proceeded by establishing as to whether they possessed the defining hNSCs feature known as multipotency,i.e. the ability to generate all the three major neural cell types: neurons, astrocytes and oligodendrocytes [25]. To do so, we analyzed both bulk population cultures as well as three clonal cell lines derived from the former by means of standard limiting dilution techniques [27]. Differentiation of T-IhNSCs was triggered as typically done with wild type hNSCs, i.e. by growth factor removal and was investigated using antibodies against lineage-specific, pan-neural antigens. Anti-MAP5 (Figure S2), anti-βIII-tubulin and anti-microtubule associated protein 2 (MAP2) were used to label neurons, anti-vimentin (Figure S2) and anti-GFAP served to detect astrocytes, and anti-O4 (Figure S2) and anti-GalC marked oligodendrocytes.

**Figure 2:**
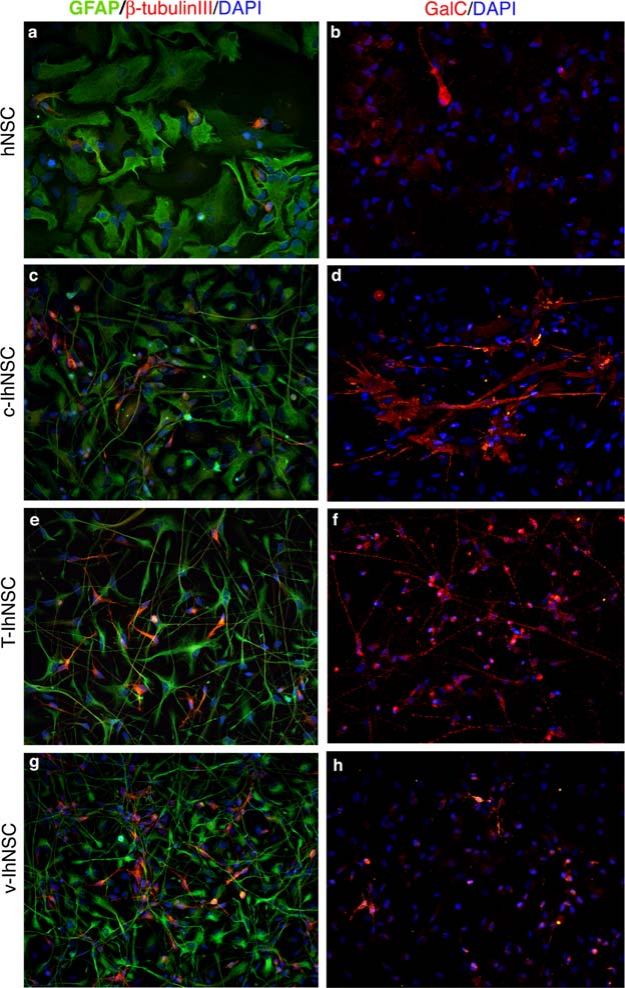
Multipotency. Cells from hNSC, c-IhNSC, T-IhNSC and v-IhNSC cells line were differentiated on laminin-coated glass coverslips in the absence of growth factors (see materials and methods) for 10 (a,c,e,g) and 17 days (b,d,f,h). Immunofluorescence staining showing the presence of astrocytes (GFAP+, green, a,c,e,g), neurons (β-tubulin+, red, a,c,e,g) and oligodendrocytes (GalC+, red, b,d,f,h). The three major neural lineage markers were detected in distinct cells of the same cell population of origin, thus demonstrating the multipotency of IhNSCs. DAPI nuclear staining (blue) is also shown to detect total cells. Magnification 20×.

As observed in parental hNSCs (Fig. 2a–b, Figure S2a–c), c-IhNSCs (Fig. 2c–d, Figure S2d–f) and v-IhNSCs (Fig. 2g–h, Figure S2l–n), both bulk cultures and clonally expanded T-IhNSC (Fig. 2e–f, Figure S2g–i) cells retained the ability to give rise to the three main neural cell types. Interestingly, a double immunofluorescence analysis of T-IhNSC-derived progenitors (3div in FGF2) with anti-βIII-tubulin and anti-GFAP showed the sporadic occurrence of asymmetric divisions (n<0,1% over the total number of cell divisions, given a frequency of cell divisions  = 0.87±0.092% over the total cell number) in the differentiating culture (Figure S3, panel B). With regard to neuronal differentiation, unipolar neurons were recognizable in the T-IhNSC culture by day 10^th^, which extended long, fine processes, bearing varicosities by day 17^th^ (Figure S4). This morphology resembled that of v-IhNSC differentiating cells (Figure S4l-n) [9].

**Figure 3:**
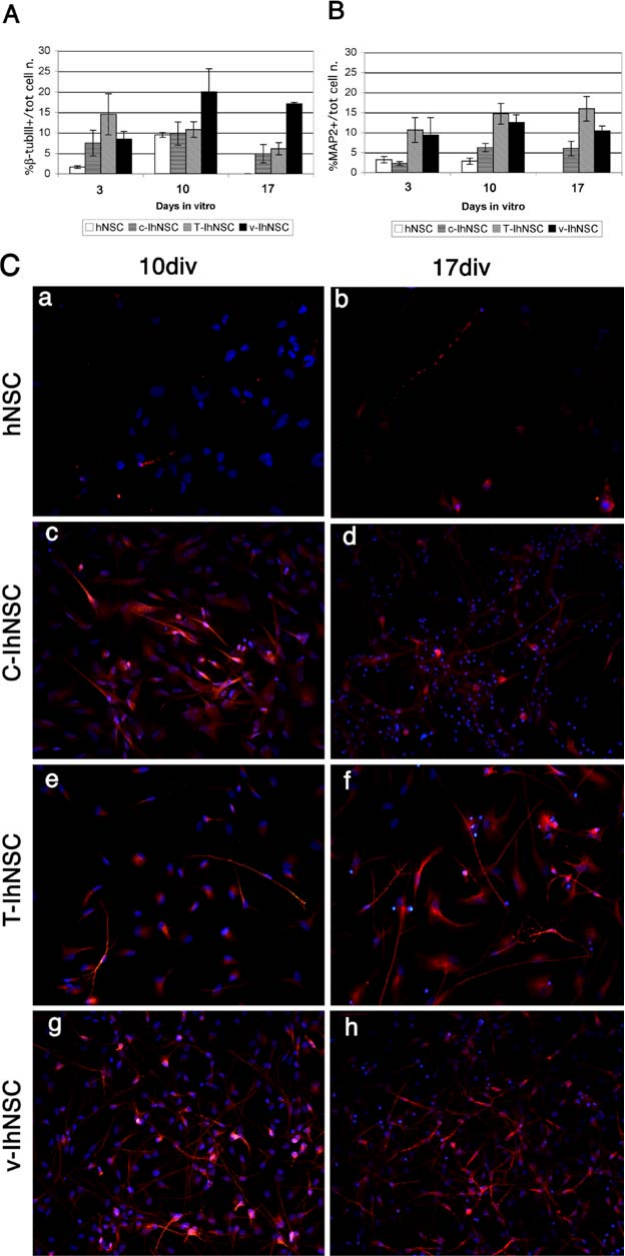
Quantitative analysis of neuronal markers expression during differentiation. A and B) Quantitative analysis of the neuronal differentiation capacity of h-NSC, c-IhNSC, T-IhNSC and v-IhNSCs cells. Cells were differentiated in adhesion on laminin in the absence of mitogenic factors and analyzed at 3, 10 and 17 days. Data are expressed as percentages of β-tubulinIII (early neuronal marker) and MAP2+ (which is expressed later during neuronal differentiation). Magnification 20×. Panel C: Immunofluorescence of hNSC (a and b), c-IhNSC (c and d), T-IhNSC (e and f) and v-IhNSCs (g and h) cell line for MAP2 at 10 (a,c,e and d) and 17 (b,d,f and h) days in vitro. DAPI nuclear staining (blue) is also shown to detect the total cell number. Magnification 20×.

At day 10, βIII-tubulin-IR cells generated by T-IhNSCs peaked at 10.78±1.89% of the total cell number (Fig. 3A), which was comparable to parental cells (10.1±0.43%) [25] or c-IhNSCs (9.79 ±2.83%) but significantly lower than in v-IhNSC cultures (19.96±5.67%). To assess if this difference corresponded to a lower percentage of neurons generated by T-IhNSC as compared to v-IhNSC or, rather, to differences in the time differentiation profile, we investigated the profile of expression of the late neuronal dendritic marker MAP2. Unlike βIII-tubulin, the number of cells expressing the MAP2 dendritic protein in T-IhNSCs cultures (Fig. 3C, e and f), had already peaked at 14.68±2.57% of the total cell number at 10 DIV and this percentage was stably maintained up to 17 days in vitro (Fig. 3B) as compared with differentiated cultures from parental cells (2.82±0.79%, Fig. 3C, a and b), c-IhNSCs (6.2±1.07%)(Fig. 3C, c and d), or v-IhNSCs (12.5±1.8%) (Fig. 3C, g and h). This points to the fact that, as compared to other immortalized lines, T-IhNSCs display a differentiation profile by which the onset of the differentiation/maturation process occurs earlier. This concept was supported by findings on the astroglial differentiation in T-IhNSCs.

**Figure 4:**
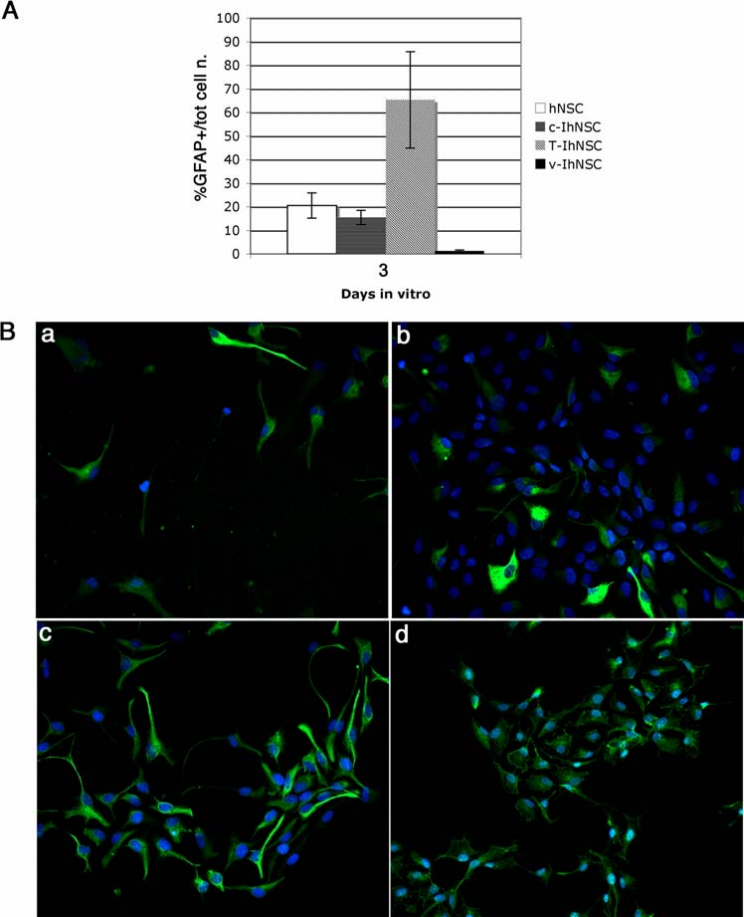
Analysis of precocious GFAP expression in T58A differentiating cells. A) Quantitative analysis of expression of the astroglial marker GFAP in progenitor cells (3 days in FGF2) from hNSC, c-IhNSC, T-IhNSC, and v-IhNSC cells. Data are expressed as percentages of GFAP+ cells of the total number of cells. B) Immunofluorescence staining of hNSC (a), c-IhNSC (b), T-IhNSC (c), and v-IhNSC (d) cells at 3 days in vitro for GFAP. DAPI nuclear staining (blue) is also shown to detect total cell number. Magnification 20×.

The astrocytes generated from T-IhNSCs were quite similar to the astrocytes generated by v-IhNSC cells. They displayed a typical protoplasmic morphology (Fig. 4B), being strictly GFAP positive and never co-labelled with βIII-tubulin (Fig. 2 a, c, e, g) – the latter phenomenon having recently been documented in transformed NSC-like glioma cells. Importantly, GFAP expression appeared significantly earlier in T-IhNSC cells as compared to the other cell lines (Fig. 4A, B). In fact, 65.3±20.45 S.E.% of the T-IhNSCs differentiated cultures (at 3 DIV) were GFAP+ as compared to 15.49±3.03 S.E.% from c-IhNSC or to 1.25±0.417 S.E.% from v-IhNSC cells or to 20.5±5.3 SE% from hNSC (Fig. 4A). A quantitative analysis of GFAP+ cells at 10 DIV was not possible, as most of the cells were GFAP+ (Fig. 2 a, c, e, f). This result is consistent with the previous observation of an early MAP2 expression onset (Fig. 3) and re-emphasizes the concept that T-IhNSCs undergo earlier differentiation and maturation as compared to v-IhNSC or c-IhNSC cells. Yet, different from parental cells and more similar to immortalized v-IhNSCs, T-IhNSC cells generate a large quantity of well-differentiated neurons, without the need of supplementing the differentiation medium with serum.

### Analysis of proliferation during differentiation

**Figure 5:**
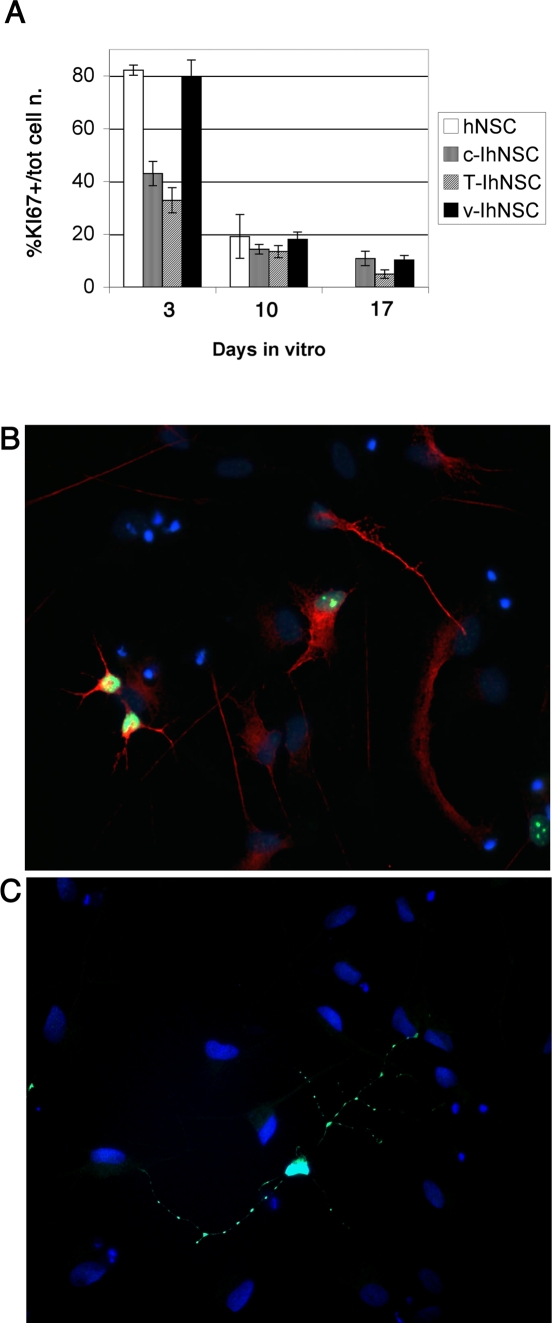
Analysis of proliferation decay during differentiation and of neuronal subtypes in T58A differentiated cells. A) hNSC, c-IhNSC, T-IhNSC and v-IhNSC cells were differentiated as described in materials and methods. The percentage of residual proliferating cells during differentiation was determined by immunofluorescence analysis with a KI67 antibody. Upon mitogen removal, T-IhNSC cells progressively cease to proliferate, reaching a terminal percentage of approximatively 4% of KI67+ cells (17 days in vitro). The number of total cells and proliferating cells were counted in five randomly chosen fields from duplicate experiments, after KI67 immunostaining. Data are expressed as percentages of KI67+ cells of the total number of cells at different times of differentiation. B) immunocolocalization of the neuronal marker β-tubulinIII with the proliferative marker KI67 in T-IhNSC cultures at 10 days of differentiation. DAPI nuclear staining (blue) is also shown. Magnification 20×. C) Exemplar neuron from T-IhNSC cultures differentiated at 17 days, fixed and immunostained with an antibody against GABA. DAPI nuclear staining (blue) is also shown. Magnification 40×.

As discussed above, a critical feature of bona fide neural stem cells is their ability to cease proliferation – except for some residual mitotic activity related to astroglial cells and late neural progenitors – and to have their progeny undergoing terminal differentiation upon growth factor removal and plating onto an adhesion substrate [25]. This trait ought to be retained by immortal hNSCs, if they are to provide a suitable, physiological model to mimic the behaviour of wild type hNSCs, while providing technical and practical advantages, such as long term and faster expandability in the absence of concomitant transformation. Hence we set out to validate this specific aspect of T-IhNSCs behaviour. Similar to their parental cells, all immortalized hNSCs did undergo growth arrest upon growth factor starvation, with scarce residual proliferation being observed. This was demonstrated by a quantitative immunofluorescence analysis performed using the proliferation marker KI67 (Fig. 5A). Once more, in good agreement with the differentiation data, suggesting that T-IhNSCs undergo early differentiation and maturation in the absence of growth factors, T-IhNSCs virtually ceased to proliferate by day 17, very similar to their parental cells [25]. At this time, only a small number of mitotic cells could be detected in both cultures (4.2±0.7% T-IhNSCs; 4.93%± 1.62 hNSCs). Notably, at the same time, growth arrest was somewhat incomplete in both v-IhNSC (10.25±1.718%) [9] and c-IhNSC cells (10.82±2.72%) (Fig. 5A). Furthermore, a significant fraction of the residual KI67+ cells in T-IhNSCs (21.68±0.33%) was a simultaneously labelled with the neuronal precursor marker β-tubulinIII (Fig. 5B), showing that a significant fraction of the residual proliferating cells were neuronal progenitors. Taken together, these findings show that T-IhNSC cells are immortalized but retain quite strict growth factor dependence and proper differentiation characteristics, which accounts for lack of transformation in our hands. This critical trait is accompanied by an apparently enhanced ability of T-IhNSC cells to generate higher percentages of neurons than their parental cells (Fig. 3 A, B).

An initial analysis of the neuronal phenotypes generated by T-IhNSCs showed that, fourteen days following the onset of differentiation, most of the total βIII-tub-IR cells co-labelled with the anti-GABA antibody (Fig. 5C). A minority of cells were glutamatergic, while no TH+ phenotype was detected in the differentiated progeny. It is worth noting how this pattern is very similar to that of the parental cells [31], re-emphasizing the resemblance of the T-IhNSC cells to wild-type hNSCs.

### T58A cells as a source of mature oligodendrocytes

**Figure 6:**
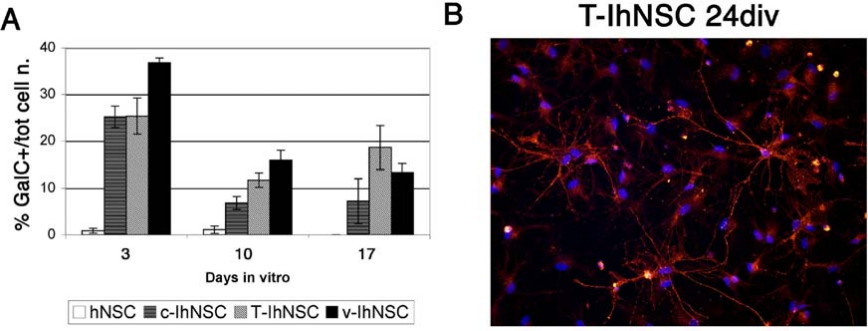
Morphological differentiation of premyelinating oligodendrocytes in differentiated T-IhNSC cells. A) Quantitative analysis of the expression of the oligodendroglial markers GalC. Data are expressed as percentages of positive cells over total number of cells at 3, 10, 17 days of differentiation. B) Exemplar oligodendrocytes obtained by T-IhNSC cells differentiation at 24 days in vitro. The oligodendroglial cells were labelled with GalC. DAPI nuclear staining (blue) is also shown. Magnification 40×.

A perusal of the literature on hNSCs, readily underlines how one of the major hurdles in this area has been to obtain the differentiation of hNSCs into a meaningful and useful amount of oligodendrocytes [31]–[34]. Strikingly, the T-IhNSC cells described here, display a unique capacity to generate bona-fide oligodendrocytes, which rarely appear in differentiated parental cultures. The genesis of numerous cells immunoreactive for GalC in T-IhNSC was readily apparent by 17 days after removal of mitogenis. At this time, oligodendrocytes appeared as star-shaped cells, with numerous, elongated and branched processes (Fig. 2), which matured to acquire a “myelinating” morphology by 24 days in vitro (Fig. 6B). By this time (24 div) as many as 18.59±4.69% of the total cells became GalC or O4-IR versus 13.41±2.6% in v-IhNSC or 7.23±4.77 in c-IhNSC % (Fig. 6A).

These findings describe T-IhNSCs as a stable, fast-growing multipotent human NSC line cell, with an enhanced ability to generate neuronal and oligodendroglia cells which, in a way heretofore unprecedented, preserve a behaviour strictly reminiscent of wild-type hNSCs.

## Discussion

Renewable and stable sources of human brain cells, bearing all of the features of multipotent neural stem cells, provide an invaluable tool in basic and preclinical studies in neuroscience [35]. In this context, we recently reported the establishment of v-myc, conditionally immortalized, human neural stem cell lines, which retain strict mitogen dependence in culture and generate mature neuronal and glial progeny including, for the first time, a significant percentage of oligodendroglial cells. [5].

In considering that v-myc is a well-known super-oncogene, bearing different mutations of the c-myc gene at multiple sites, we set out to find an alternative, immortalizing agent that retained a structure closer to the wild-type allele. The c-myc T58A gene bears a single mutation as compared to wild type c-myc and has never been used before to immortalize cells. The T58A mutation has been identified in several cases of Burkitt's lymphomas. It is involved in the stabilization of c-myc, likely by impairing the rapid proteolysis of c-myc through Thr-58 phosphorylation [17]. Here, we report the first successful attempt to use c-myc T58A to establish an immortal cell line from human neural stem cells of fetal origin. The resulting cell line (T-IhNSC) has the capacity to generate unlimited quantities of conditionally immortalized hNSCs, which can then be differentiated into mature neurons, astroglia and significant numbers of oligodendroglial cells. The characteristics of this cell line were compared to their wild type human neural stem cells and to hNSCs immortalized with c-myc wt or v-myc [9], using the same source of fetal neural stem cells. This allows to determine any similarities and potential advantages of T-IhNSCs, in addition to this line having been established using a much lesser mutated and better characterized c-myc mutant.

Our first conclusion was that T-IhNSC cells preserved all of the characteristics of their parental hNSCs while, at the same time, expressing a significantly faster kinetic expansion rate. the extended proliferation and self-renewal capacity of this cell line makes it amenable for at least 120 subculturing steps. This was reflected in a significantly shorter, average doubling time, than that of c-IhNSCs (Figure S1) or wild type hNSCs [25] and slightly longer than v-IhNSCs. Accordingly, while an average 25% of the cells was engaged in S phase in all three immortalized cell lines, the T-IhNSC cell line displayed a clonogenic potential similar to v-IhNSCs and significantly higher than c-IhNSC cell line or parental hNSCs [25], [30]. This finding points to an increased viability and self-renewal ability in T-IhNSCs and is in agreement with studies from Cartwright et al. [12], showing that c-T58A promotes self-renewal and maintenance of pluripotency in ES cells and that the T58A allele in Burkitt's lymphomas confers reduced apoptotic activity [13], [16].

To date, T-IhNSC have been propagated for up to 120 passages without any sign of senescence or proliferative decay or transforming activity, which satisfies one of the requirements for their classification as immortalized cell lines.

Consistent with the view that such immortalization was conditional in its own nature, proliferation and self-renewal of T-IhNSC remained strictly growth factor dependent, as shown previously for normal hNSCs (Fig. 1B, 5A). Upon growth factor removal, T-IhNSC underwent growth arrest and differentiation and ceased divisions (Fig. 5A).

These findings show that, in association with a lower expansion rate as compared to v-IhNSCs, T-IhNSCs display an earlier, safer differentiation profile. In good keeping with the current view that that T58A myc does not seem to act as an oncogene in brain cells, these observations also provide support to the non-transformed nature of the T-IhNSCs.

The latter was confirmed by the results of a soft-agar anchorage independent colony formation assay in which, neither v-IhNSC nor T-IhNSC or wt-IhNSC lines produced any colony, whereas glioblastoma cells did so, as expected (Figure S5). In addition, the intraparenchimal injection of T-IhNSC cells into SCID mice brains, up to six months after transplantation, yielded no tumor formation (data not shown). Although broader studies are required to prove this point beyond any reasonable doubt, previous findings cogently support this view. We have recently shown that, not even v-IhNSCs could induce tumor formation when transplanted into the brain parenchyma or intravenously into immunodeficient mice [9], [35]. Thus, if transformation does not take place when using the v-myc gene, it is not unexpected that the c-myc T58A gene that we used to establish T-IhNSCs, will be even less likely to bring about transformation in human neural stem cells. In fact, unlike the c-myc T58A gene that contains a single mutation and no proven tumorigenic capacity, the v-myc role in oncogenesis has been cogently shown in fibroblast and myeloid cells [23], [24]. Further, it has been proven that v-myc is much more effective than c-myc in eliciting transformation, due to combinatorial effects of 5′ and 3′ mutations [36].

Another important feature of T-IhNSCs is that they retain the multipotency typical of their parental cells, however with an advantageous improved capacity to generate more neurons and oligodendroglia, similar to v-IhNSCs. A review of the properties of previously established immortalized cell lines and of their differentiation capacity shows that these lines generate neurons and astroglial [5]. However, differentiation in these cells appeared to be regulated by cues other than those normally observed in normal human neural cells [31]. Unlike these lines, and similar to normal hNSCs and v-IhNSCs [9], differentiation was a spontaneous event that ensued in T-IhNSCs upon growth factor starvation. The overall amount of neurons and astroglia generated by T-IhNSCs and v-IhNSCs were comparable and significantly higher with respect to c-IhNSC cells and hNSC cells (Fig. 3 A,B; Fig. 6A). Of note, these neurons expressed the appropriate antigenic markers MAP5, β-tubulin III and MAP2 and never colocalized with GFAP inside the same cells (Fig. 2 a, c, e, g; Figure S2 a, d, g, l) – the latter being a likely sign of transformation [30].

As hoped, T-IhNSC-derived neurons appeared to undergo progressive maturation, spreading out long processes with varicosities typical of mature dendrites, as shown previously for v-IhNSCs.

A preliminary characterization showed that the neuronal neurotransmitter phenotype most represented amongst the T-IhNSCs progeny was the GABAergic one, which appears to be a somewhat default phenotype for cultured neural stem cells [31]. We are currently investigating if, similar to hNSCs, also T-IhNSCs can be induced to generate catecholaminergic cells by exposure to specific extracellular cues [31].

Regarding the ability to give rise to cells of the glial lineage, the ability to give rise to *bona fide* oligodendrocytes is a pivotal feature in cell lines that are applicable in the study of human neurogenesis, in drug discovery and for modeling cell therapy for demyelinating disorders. In this work we show that T-IhNSC display a vastly increased capacity to give rise to human oligodendrocytes (up to 25.1% of the total cell number) that not only exceed that of their parent hNSC cells (1–4% of the total cells; [25]) but is even significantly greater of that observed in v-IhNSCs or c-IhNSCs (16.1% and 12% of the total cells, respectively). Furthermore, the morphological maturation of oligodendrocytes from T-IhNSC cells, occurred as early as 17 days from the onset of differentiation (as compared to 24 div in vIhNSC[9]), with the morphological features of these cells approximating that of a fully mature oligodendrocyte better than in any other cell (Fig. 2, b, d, f, h, Fig. 6B) This makes T-IhNSC the cell line with the highest oligodendroglial developmental potential to become available, to date.

### Conclusions and remarks

In conclusion, our findings describe a new, continuous cell line of human multipotent neural stem cells, that have been conditionally immortalized using a mutant of c-myc never used in such an endeavour before. Given that this gene has no known oncogenic ability and bears a single, well-charaterized mutation as compared to the standard immortalizing agent v-myc, the risk of transformation of the targeted hNSCs is reduced. In fact, T-IhNSCs display no transformation, but bear interesting characteristics, including rapid expansion in culture, retention of growth-factor dependence, multipotentiality and stability over time. The complement of functional features of T-IhNSCs are intermediate between those of previously established v-IhNSCs and normal hNSCs. This provides a novel model for the development of high throughput assays for both basic and therapeutic studies on human neural cell development and a valid human alternative to rodent cell lines for the identification of neuroactive molecules targeting neurons and oligodendroglia.

The genuine multipotentiality of immortalized cells, their strict growth factor dependence, the early appearance of differentiation markers, and their rapid proliferation arrest after differentiation, favours the T-IhNSC cell line for future clinical application studies. In addition, T-IhNSCs may enable preliminary, broad screening studies that could be subsequently confirmed and refined by utilizing normal hNSCs. This represents a step towards elucidating many unanswered questions in developmental human neurobiology.

## Supporting Information

Figure S1 Proliferation and cell-cycle analyses. (A–D) BrdU incorporation assay of in hNSC (A), c-IhNSC (B), T-IhNSC (C) and v-IhNSC (D) cells. A pulse of BrdU was given to IhNSC cells for 20 min and then the percentage of cells in S-phase was identified by cytofluorimetric analysis. Actively proliferating cells (in S-phase) are shown in the chart as the percentage of the population BrdU+. (E) Analysis of the cell cycle duration showing the relative time lengths (hours) of the different phases.
(5.80 MB TIF)

Figure S2 Analysis of neuronal and glial markers expression during differentiation. Immunofluorescence of hNSC (a–c), c-IhNSC (d–f), T-IhNSC (g–i) and v-IhNSCs (l–n) cell line, showing expression of cells lineage specific markers in cells differentiated for 10 days in adhesion on laminin in the absence of growth factors. Astroglial cell marker GFAP is shown in green (a,d,g,l,c,f,i,n). The neuronal markers MAP-5 is shown in red (a,d,g,l) and β-tubulinIII in green (b,e,h,m). Astrocytes precursor marker, Vimentin, is shown in red (b,e,h,m) and the oligondendrocytes marker, O4 is shown in red (c,f,i,n). DAPI nuclear staining (blue) is also shown to detect cells nuclei. Magnification 40×.
(7.36 MB TIF)

Figure S3 Asymmetric and symmetric divisions of T58A progenitor cells. Panel A-B: immunofluorescence analysis of T-IhNSC cells after 3 days in culture with FGF2 for glial (GFAP in green), neuronal (β-tubulinIII in red). DAPI nuclear staining (blue) is also shown. Panel A shows a symmetric division of a neuronal unipotent progenitor. Panel B shows the segregation of β-tubulinIII and GFAP in a bipotent neuroglial progenitor.
(10.49 MB TIF)

Figure S4 The differentiation potential of T58A stem cells. Panel a–n: phase-bright microphotographs of hNSC (a–c), c-IhNSC (d–f), T-IhNSC (g–i) and v-IhNSCs (l–n) cells attached to a laminin-treated surface. Freshly dissociated neurospheres (a,d,g,l) were cultured for 3 days in the presence of FGF2 (b, e, h and m) and teminally differentiated in the absence of mitogenic factors (c, f, i and n).
(4.09 MB TIF)

Figure S5 Soft agar colony formation assay. IhNSC (A) cells were seeded as a single cell suspension (1250 cells/well in a 24-wells culture dish) in soft agar (0.4% in growth medium) and incubated at 37°C, 5% CO2 as requested in the “Cell Transformation Detection Assay” kit (CHEMICON n. ECM570). Cells were analyzed using the cell stain solution included with the kit to identify cell colony formation after 3 weeks. We could not detect cells colonies in wells containing IhNSCs grown in these conditions. A glioblastoma cell line (GBM) was used as positive control. GBM cells proliferated and produced large clusters (B, 10.3±2.8 colonies/well).
(0.88 MB TIF)
