# Supplementary figures and images for: Immortalization of Human Neural Stem Cells with the c-Myc Mutant T58A
Source: PLoS One. 2008 Oct 2;3(10):e3310. doi: 10.1371/journal.pone.0003310 (PMC2561001; doi:10.1371/journal.pone.0003310)

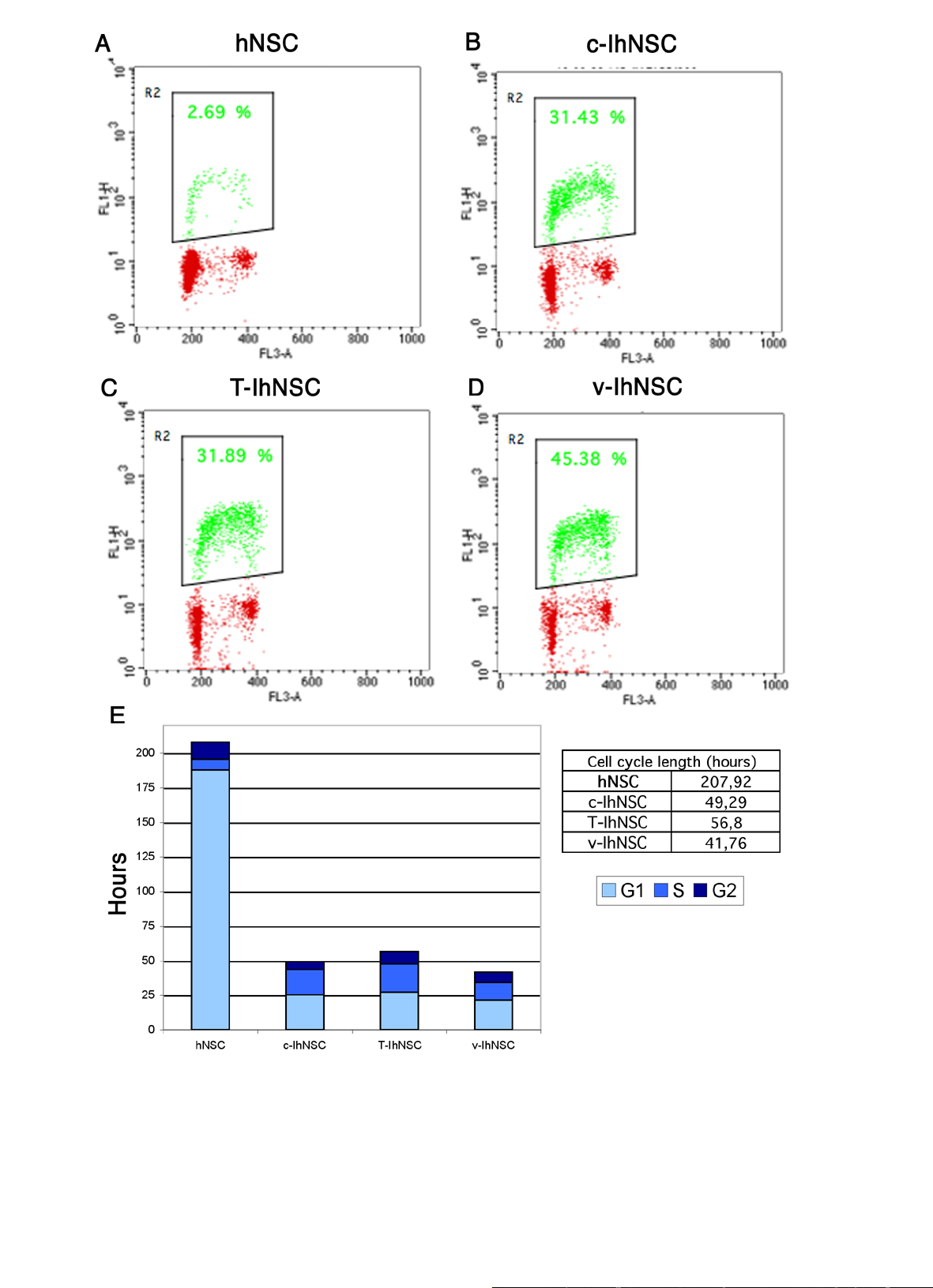

Supplement: Figure S1 — Proliferation and cell-cycle analyses. (A–D) BrdU incorporation assay of in hNSC (A), c-IhNSC (B), T-IhNSC (C) and v-IhNSC (D) cells. A pulse of BrdU was given to IhNSC cells for 20 min and then the percentage of cells in S-phase was identified by cytofluorimetric analysis. Actively proliferating cells (in S-phase) are shown in the chart as the percentage of the population BrdU+. (E) Analysis of the cell cycle duration showing the relative time lengths (hours) of the different phases. (5.80 MB TIF) [file pone.0003310.s001.tif]

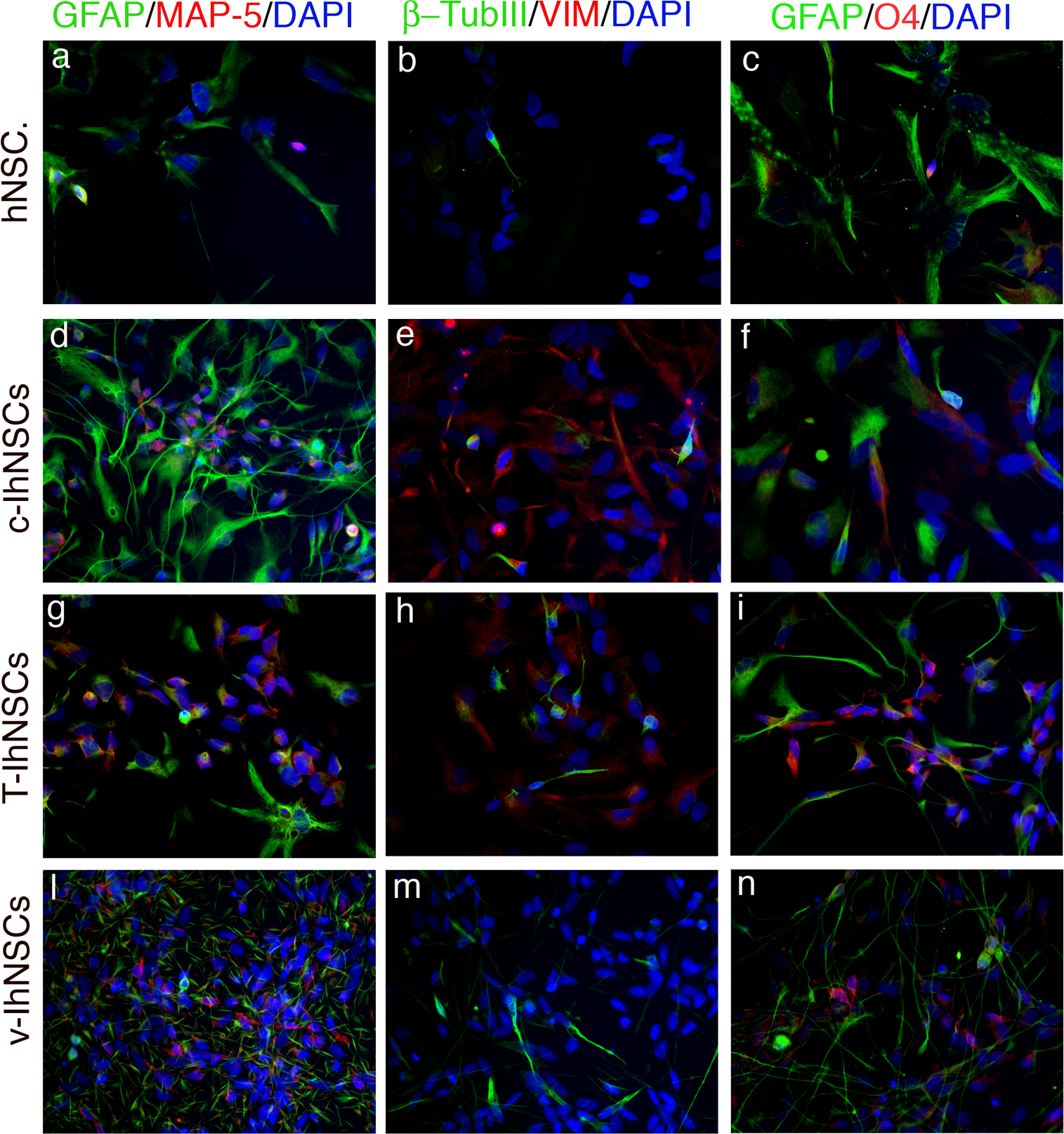

Supplement: Figure S2 — Analysis of neuronal and glial markers expression during differentiation. Immunofluorescence of hNSC (a–c), c-IhNSC (d–f), T-IhNSC (g–i) and v-IhNSCs (l–n) cell line, showing expression of cells lineage specific markers in cells differentiated for 10 days in adhesion on laminin in the absence of growth factors. Astroglial cell marker GFAP is shown in green (a,d,g,l,c,f,i,n). The neuronal markers MAP-5 is shown in red (a,d,g,l) and β-tubulinIII in green (b,e,h,m). Astrocytes precursor marker, Vimentin, is shown in red (b,e,h,m) and the oligondendrocytes marker, O4 is shown in red (c,f,i,n). DAPI nuclear staining (blue) is also shown to detect cells nuclei. Magnification 40×. (7.36 MB TIF) [file pone.0003310.s002.tif]

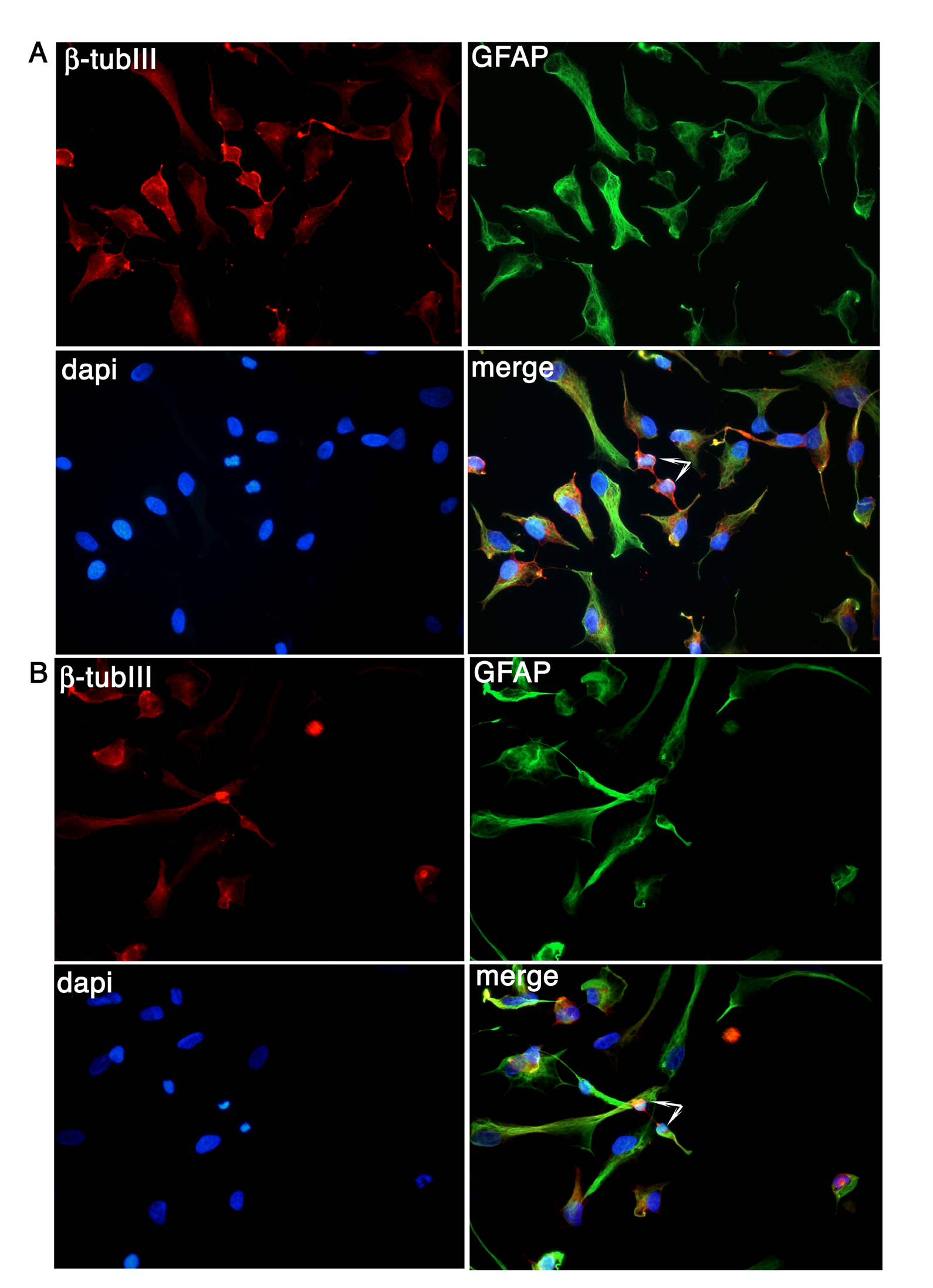

Supplement: Figure S3 — Asymmetric and symmetric divisions of T58A progenitor cells. Panel A-B: immunofluorescence analysis of T-IhNSC cells after 3 days in culture with FGF2 for glial (GFAP in green), neuronal (β-tubulinIII in red). DAPI nuclear staining (blue) is also shown. Panel A shows a symmetric division of a neuronal unipotent progenitor. Panel B shows the segregation of β-tubulinIII and GFAP in a bipotent neuroglial progenitor. (10.49 MB TIF) [file pone.0003310.s003.tif]

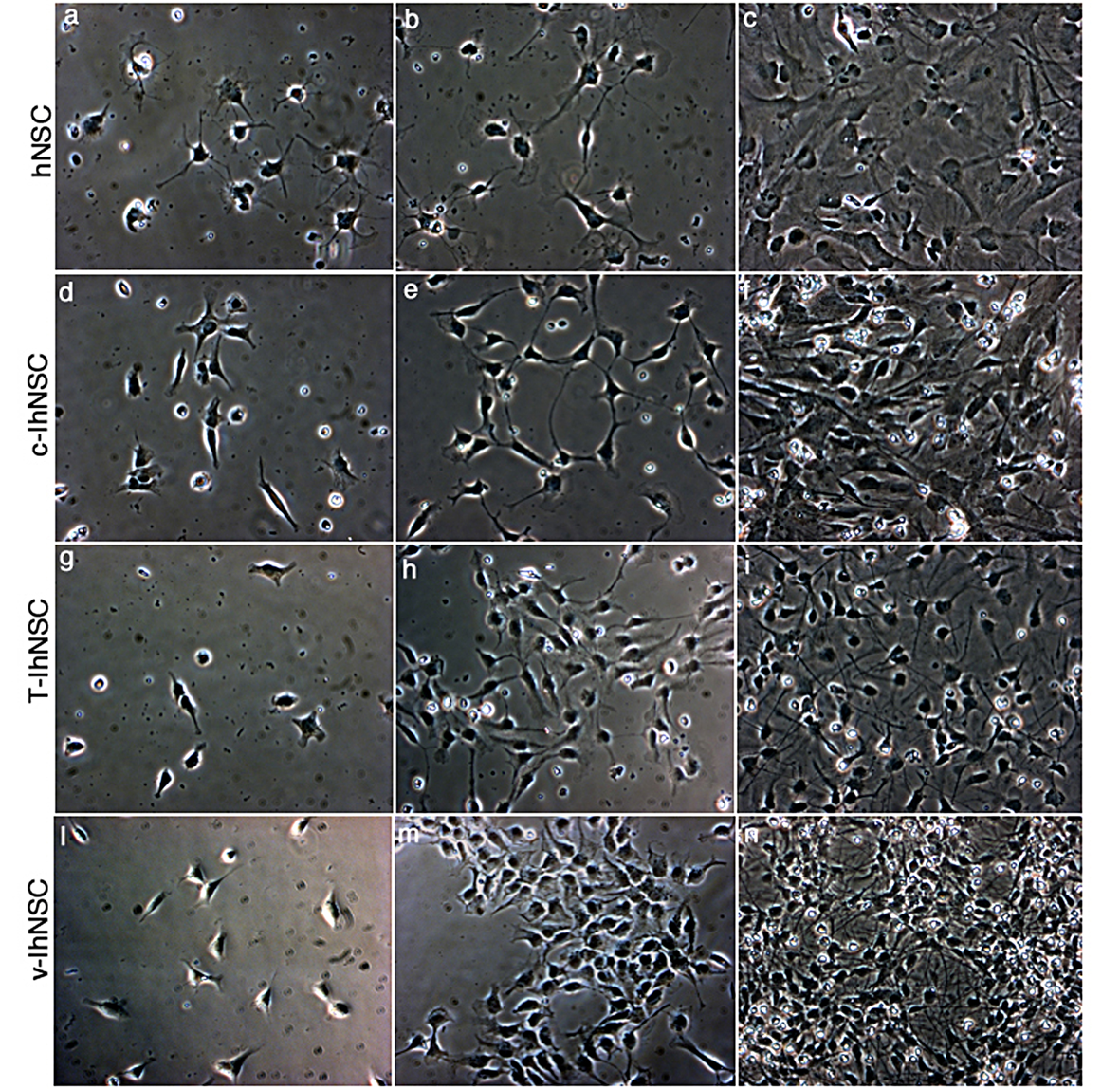

Supplement: Figure S4 — The differentiation potential of T58A stem cells. Panel a–n: phase-bright microphotographs of hNSC (a–c), c-IhNSC (d–f), T-IhNSC (g–i) and v-IhNSCs (l–n) cells attached to a laminin-treated surface. Freshly dissociated neurospheres (a,d,g,l) were cultured for 3 days in the presence of FGF2 (b, e, h and m) and teminally differentiated in the absence of mitogenic factors (c, f, i and n). (4.09 MB TIF) [file pone.0003310.s004.tif]

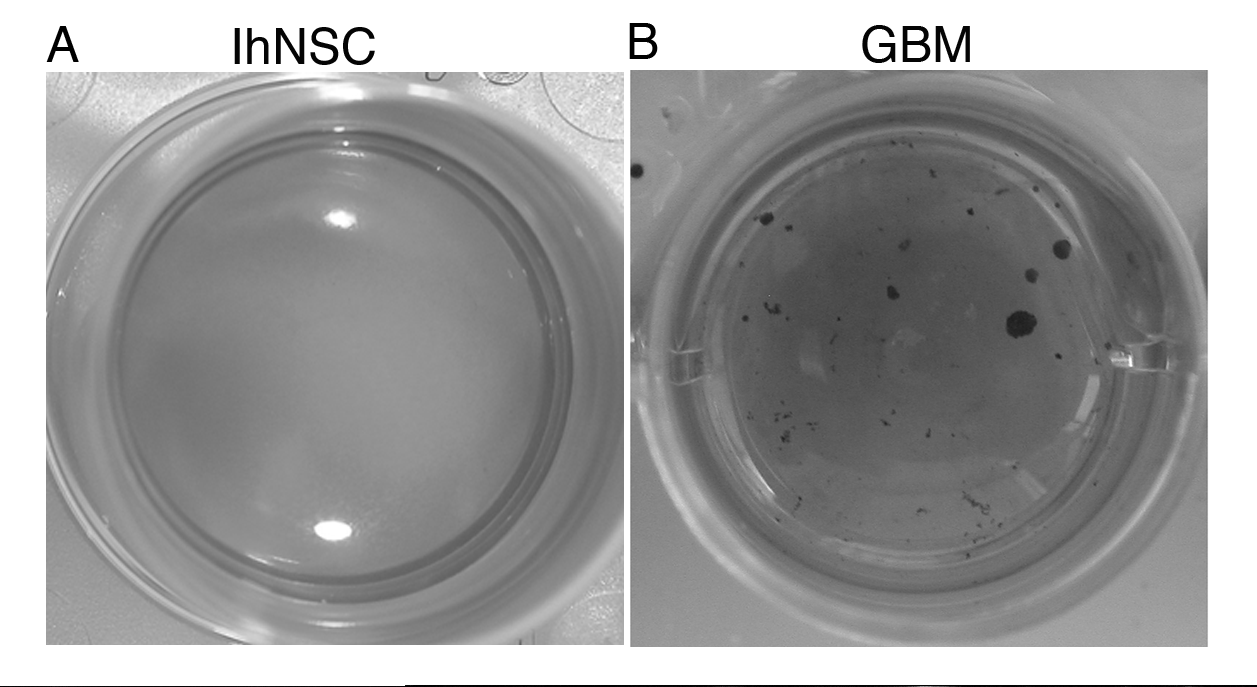

Supplement: Figure S5 — Soft agar colony formation assay. IhNSC (A) cells were seeded as a single cell suspension (1250 cells/well in a 24-wells culture dish) in soft agar (0.4% in growth medium) and incubated at 37°C, 5% CO2 as requested in the “Cell Transformation Detection Assay” kit (CHEMICON n. ECM570). Cells were analyzed using the cell stain solution included with the kit to identify cell colony formation after 3 weeks. We could not detect cells colonies in wells containing IhNSCs grown in these conditions. A glioblastoma cell line (GBM) was used as positive control. GBM cells proliferated and produced large clusters (B, 10.3±2.8 colonies/well). (0.88 MB TIF) [file pone.0003310.s005.tif]
